# Protective role of selenium in sepsis: Mechanisms and potential therapeutic strategies

**DOI:** 10.1515/med-2025-1296

**Published:** 2025-10-29

**Authors:** Yukun Liu, Xuan Zhao, Zhikai Xu, Zhanfei Li, Yuchang Wang

**Affiliations:** Department of Plastic and Aesthetic Surgery, Tongji Hospital, Tongji Medical College, Huazhong University of Science and Technology, Wuhan, 430030, China; Division of Trauma Surgery, Emergency Surgery & Surgical Critical, Tongji Hospital, Tongji Medical College, Huazhong University of Science and Technology, Wuhan, 430030, China; Trauma Center, Tongji Hospital, Tongji Medical College, Huazhong University of Science and Technology, Wuhan, 430030, China; Department of Emergency and Critical Care Medicine, Tongji Hospital, Tongji Medical College, Huazhong University of Science and Technology, Wuhan, 430030, China; Sino-German Research Institute of Disaster Medicine, Wuhan, 430030, China

**Keywords:** sepsis, selenium, selenoprotein, multiple organ dysfunction syndrome, organ dysfunction, organ damage

## Abstract

Sepsis is an inflammatory disease caused by a severe infection, and its pathological process involves complex immune reactions and inflammatory cascades. This condition often leads to multiple organ dysfunction syndrome, which is one of the main causes of patient mortality. In recent years, researchers have paid extensive attention to the protective role of selenium (Se) in sepsis. Se is believed to potentially counteract organ dysfunction caused by sepsis through various mechanisms and is considered a potential therapeutic strategy. This review extensively discusses the potential mechanisms of Se in sepsis. We explore the antioxidant and anti-inflammatory properties of Se, as well as its regulatory effects on immune cell activity, expression of inflammatory mediators, and oxidative stress. In addition, we examine the impact of Se on organ damage and organ dysfunction caused by sepsis, with a focus on its protective effects on important organs such as the cardiovascular system, respiratory system, kidneys, and liver. We evaluate relevant preclinical and clinical studies to assess the potential of Se as a treatment for sepsis-related organ dysfunction. We discuss the optimization of Se administration routes, dosages, and timing, and summarize the impact of Se on clinical outcomes and survival rates. In summary, Se demonstrates significant potential as a therapeutic strategy in sepsis-related organ dysfunction. However, further research is still needed to delve into the mechanisms of Se and optimize its application in clinical practice. This will provide breakthroughs in the treatment of sepsis patients, improving their prognosis and survival rates.

## 1 Introduction

Sepsis is a severe infectious disease characterized by the occurrence of multiple organ dysfunction, leading to high mortality rates and poor prognosis [1,2]. Globally, the annual incidence of sepsis is estimated to be approximately 276–678 cases per 100,000 persons, with mortality rates reaching 22.5–26.7%. The mortality is even higher among patients with severe sepsis or septic shock [3]. The causative pathogens of sepsis are diverse, encompassing bacteria, viruses, fungi, and other microorganisms, and variations in pathogen type and site of infection significantly influence disease progression and prognosis [4]. Despite advances over recent decades in sepsis management – including antimicrobial therapy, fluid resuscitation, and organ support – overall clinical outcomes remain unsatisfactory, and treatment continues to face substantial challenges [5–8]. Consequently, the development of novel therapeutic strategies has become a critical focus in contemporary sepsis research.

Oxidative stress and mitochondrial dysfunction are pivotal mechanisms in the pathogenesis of sepsis [9]. During sepsis, excessive production of reactive oxygen species (ROS) and reactive nitrogen species damages mitochondrial membrane structures and the electron transport chain, leading to energy metabolism imbalance, increased apoptosis, and organ dysfunction [10]. Moreover, mitochondrial injury can trigger the release of mitochondrial DNA and inflammatory mediators, thereby further activating the host immune response and exacerbating systemic inflammatory response syndrome [11]. Selenium (Se), as an essential trace element, is widely recognized for its various biological functions and protective effects [12,13]. Se can participate in the regulation of biological processes such as inflammation, immune function, and oxidative stress through multiple mechanisms, thereby playing a regulatory role in the development of sepsis and organ damage [14–16]. In recent years, an increasing number of studies have suggested that Se may have potential protective effects in sepsis, alleviating organ dysfunction and improving patient prognosis [17,18]. Recent studies have revealed that, in the early stages of sepsis, excessive ROS and peroxynitrite induce endothelial barrier disruption and excessive leukocyte activation, accompanied by a rapid decline in selenoprotein P (SePP) levels. Notably, low concentrations of sodium selenite (Na₂SeO₃) can promote SePP synthesis, whereas high concentrations exert selective cytotoxicity toward leukocytes. The combined application of these effects may represent a promising strategy for early intervention in sepsis [19].

In this review, we will systematically explore the potential mechanisms and therapeutic significance of Se in sepsis. We will provide a detailed elucidation of the biological functions and metabolic pathways of Se in the human body, with a focus on its role in regulating inflammation and immune function, particularly in the context of oxidative stress and immune imbalance caused by sepsis. We will delve into the regulation of inflammatory mediators, the impact on cell signaling pathways, and the modulation of immune cell function by Se. Furthermore, we will examine the protective role of Se in sepsis-related organ dysfunction through its effects on mitochondrial function, attenuation of cellular apoptosis, and reduction in cell damage. The protective effects in important organs such as the nervous system, cardiovascular system, respiratory system, kidneys, and liver will be discussed, along with their potential molecular mechanisms. Finally, we will evaluate relevant preclinical and clinical studies to assess the potential of Se as a treatment for sepsis-related organ dysfunction. Through a comprehensive exploration in this review, we aim to provide a foundation for further investigating the mechanisms and clinical applications of Se in sepsis, and to inspire the development of new treatment strategies. This will contribute to improving the prognosis of sepsis patients and provide new breakthroughs for clinical practice.

## 2 Se and selenoproteins in health

Se is an essential trace nutrient that was first discovered by Swedish chemist Jöns Jacob Berzelius in 1817. Initially considered a naturally occurring toxic substance [20], the perception of Se as a health threat changed when Schwarz and Foltz accidentally discovered its ability to prevent liver necrosis in rats in 1957 [21]. Since then, Se has been recognized for its important role as a cofactor for more than 30 selenoproteins involved in maintaining basic physiological functions [22]. Se’s physiological functions are primarily manifested through selenoproteins, a group of proteins with diverse biological functions, particularly related to cell signaling and antioxidant responses, thyroid hormone metabolism, and humoral and cellular immune reactions [23]. Approximately 60% of serum Se is converted into SePP, 30% is converted into GSH-Px, and 5–10% is converted into albumin [22]. The GSH-Px family catalyzes the reduction of various hydroperoxides and synergistically contributes to the antioxidant defense against lipid peroxidation along with vitamin E [24].

In terms of physiological effects, Se is an integral component of various selenoenzymes, including glutathione peroxidases (GPxs) [25]. These enzymes participate in regulating intracellular redox reactions, neutralizing free radicals and oxidative substances, and protecting cells from oxidative damage [26,27]. Se, as an important antioxidant, has been reported to effectively scavenge ROS, especially hydrogen peroxide, in organs such as the heart [28], liver [29], kidneys [30], thyroid [31], and brain [32]. Additionally, Se plays a crucial role in maintaining normal immune system function. It contributes to the proliferation, activation, and regulation of immune cells, promoting their normal functionality and preserving immune system balance [26,27,33–37]. Se also plays a critical role in thyroid hormone synthesis and metabolism. It participates in the conversion and regulation of thyroid hormones, maintaining normal thyroid function and metabolic rate [38–40].

**Figure 1:**
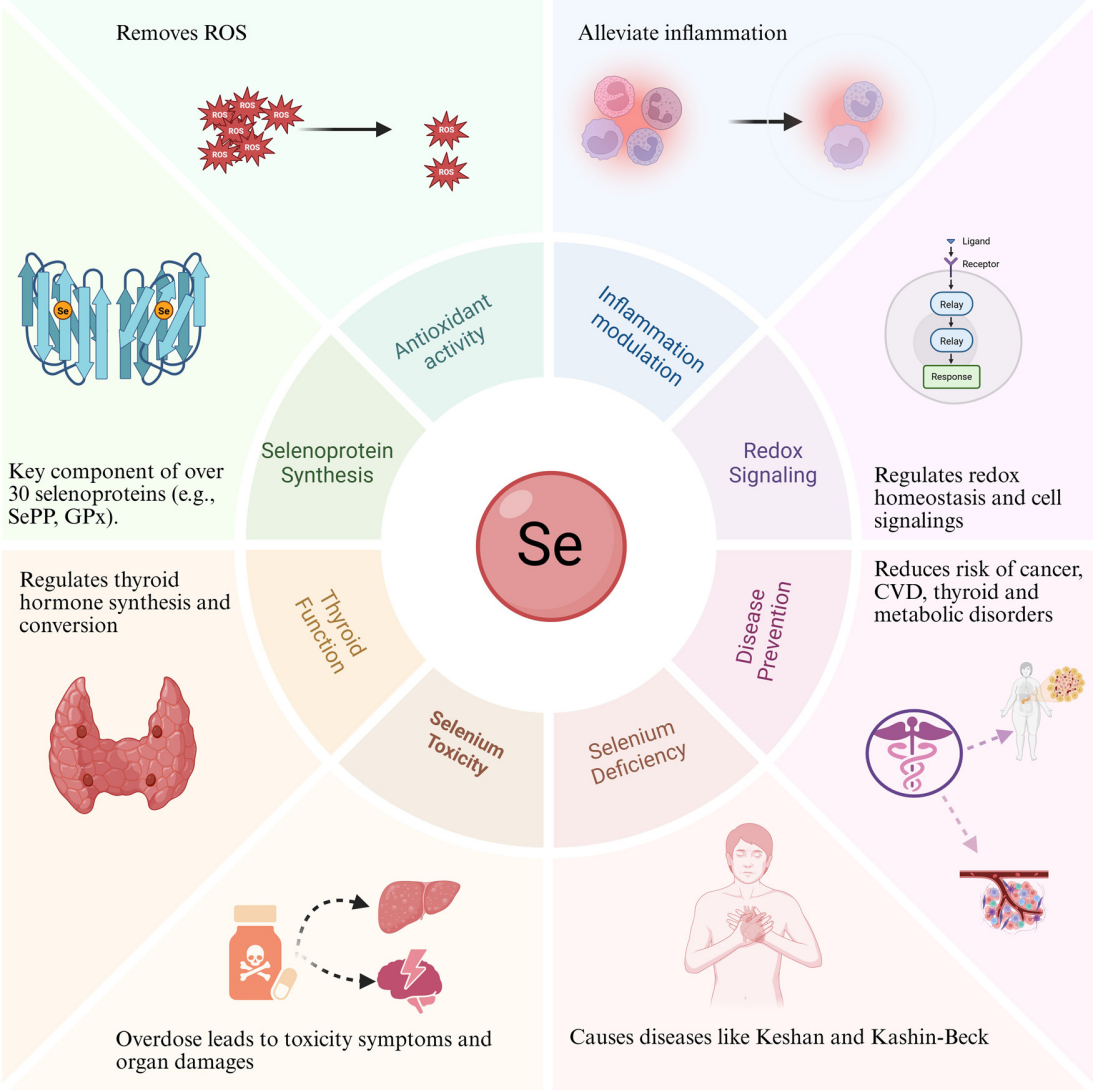
Multifaceted biological roles and health implications of Se. Se exerts diverse physiological functions through its incorporation into selenoproteins such as GPx and SePP. These functions include antioxidant activity by scavenging ROS, modulation of inflammation, regulation of redox signaling pathways, and maintenance of thyroid hormone metabolism. Se deficiency is linked to disorders such as Keshan disease and Kashin-Beck disease, while Se toxicity due to overdose can lead to organ damage. Adequate Se intake contributes to disease prevention, reducing the risk of cancer, CVD, thyroid dysfunction, and metabolic disorders.

Long-term Se deficiency can lead to Se deficiency diseases, including Keshan disease and Kashin-Beck disease. Se deficiency affects the functioning of the cardiovascular, immune, and nervous systems, resulting in pathological changes such as cardiac damage, muscle pain, and impaired immune function. Se deficiency is associated with increased risk of thyroid diseases, cardiovascular diseases (CVD), immune system disorders, and certain cancers. Conversely, excessive Se intake can lead to Se toxicity. Se toxicity is typically associated with excessive Se supplementation or abnormally high Se levels in the environment. It can cause gastrointestinal symptoms, neurological damage, liver impairment, and other pathological effects. The intake of Se and body Se levels may also be related to other diseases, such as inflammatory disorders, metabolic syndrome, and cognitive function. Adequate Se supplementation not only helps regulate the immune system but also plays a role in the modulation of brain function, CVDs, cancer, and other related conditions [39,41–43]. Thus, it provides new perspectives for the treatment of various diseases (Figure 1).

In summary, Se plays a crucial role in the physiological and pathological processes in the human body. Adequate Se intake is essential for maintaining normal physiological functions and immune system balance, while Se deficiency or excessive intake may contribute to the occurrence and development of diseases. Therefore, maintaining an appropriate level of Se intake is vital for maintaining human health.

## 3 Se and organ dysfunction in sepsis

**Figure 2:**
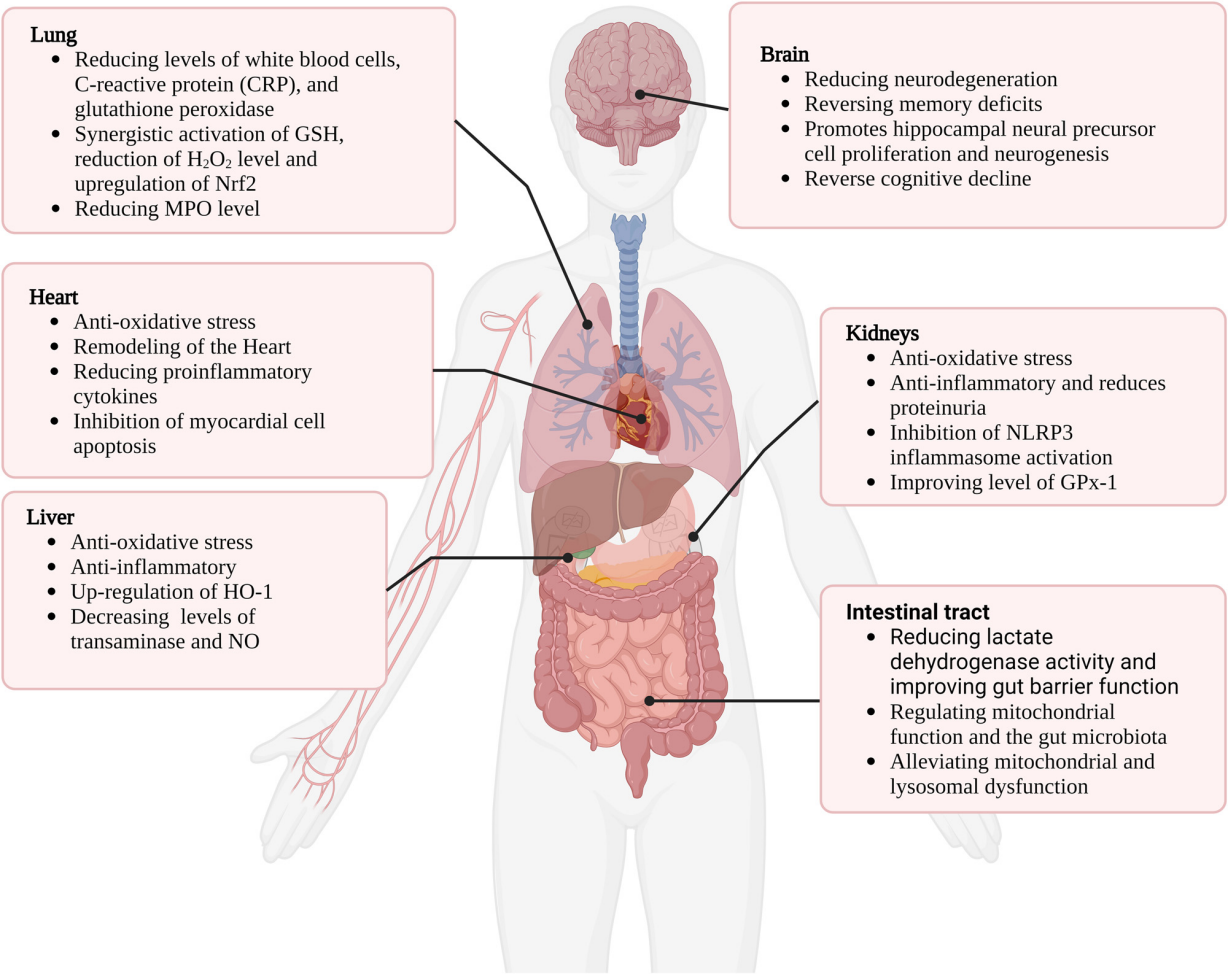
The role of Se in septic organs and its potential protective mechanisms. This schematic illustrates the organ-specific effects and protective roles of Se during sepsis. In the lungs, Se enhances antioxidant capacity and reduces inflammatory cytokine release, thereby mitigating acute lung injury. In the heart, it improves mitochondrial function and suppresses oxidative stress, contributing to the protection against septic cardiomyopathy. In the liver and kidneys, Se supports redox homeostasis, attenuates tissue damage, and helps preserve organ function. In the gastrointestinal tract, Se maintains epithelial barrier integrity and modulates immune responses. These protective effects are primarily mediated through selenoproteins, which reduce ROS, regulate redox-sensitive signaling pathways, and modulate inflammatory and immune responses. Se supplementation may thus offer therapeutic benefits by targeting multiple organ systems affected during sepsis.

Organ dysfunction caused by sepsis involves multiple organ systems, including the cardiovascular system, respiratory system, kidneys, and liver [44–48]. The pathogenesis of organ dysfunction in sepsis involves mechanisms such as inflammation, oxidative stress, cellular apoptosis, and cellular injury [49–51]. Studies have found that high-dose supplementation of Na₂SeO₃ can reduce the mortality rate in patients with severe sepsis or septic shock [52]. Understanding the potential mechanisms of Se in sepsis, including its antioxidant and anti-inflammatory properties, regulation of cellular signaling pathways, and modulation of cellular apoptosis and inflammatory mediators, can contribute to the development of therapeutic strategies for sepsis-related organ dysfunction (Figure 2).

### 3.1 Se and sepsis-related cognitive dysfunction

Sepsis can lead to acute and long-term brain dysfunction, known as sepsis-associated encephalopathy (SAE) and cognitive dysfunction. SAE refers to alterations in consciousness without direct evidence of central nervous system infection [45,53]. However, sepsis-induced cognitive dysfunction is a complex issue, and current evidence suggests that the pathogenesis is primarily characterized by systemic inflammation, blood-brain barrier dysfunction, neuroinflammation, microcirculatory dysfunction, and interactions with brain dysfunction [54,55]. Studies have shown significantly decreased plasma Se levels and erythrocyte glutathione peroxidase activity in patients with Alzheimer’s disease (AD) [56,57]. Therefore, in AD models, supplementation with exogenous Se has been found to alleviate neurodegeneration and reverse memory deficits [58,59]. Current research has discovered that Se administration promotes hippocampal neuroprogenitor cell proliferation and adult neurogenesis. Simulating the effects of exercise through dietary Se supplementation restored neurogenesis and reversed age- and hippocampal injury-related cognitive decline, suggesting potential therapeutic relevance [60]. However, there is still insufficient animal and clinical evidence to establish a direct causal relationship between Se and sepsis-related cognitive dysfunction.

### 3.2 Se and sepsis-related lung injury

Sepsis can cause lung injury and acute respiratory distress syndrome, characterized by pulmonary edema, impaired gas exchange, and respiratory distress [61,62]. Inflammatory responses and oxidative stress lead to inflammatory cell infiltration and damage to lung tissues, as well as injury to alveolar epithelial cells and pulmonary capillary endothelial cells, exacerbating lung injury and functional impairment [63,64]. A study using a rat cecal ligation and puncture (CLP) model found that Se and/or vitamin E pretreatment reduced or prevented sepsis-induced lung tissue injury, as evidenced by improved blood gas parameters (pH, pCO_2_, SaO_2_), decreased white blood cells, reduced C-reactive protein (CRP) levels, and improved glutathione peroxidase levels [65]. In another rat sepsis model, combined treatment with niacin and Se alleviated lung injury in septic patients and improved survival rates. The therapeutic effects were associated with the synergistic activation of the glutathione redox cycle, decreased hydrogen peroxide levels, and upregulation of nuclear factor erythroid 2-related factor 2 [66]. Additionally, research has found that Se treatment reduced myeloperoxidase activity in the bone marrow of mice in the CLP group, providing certain protection against lung inflammation during acute lung injury [67]. Therefore, based on current preclinical experiments, Se has potential therapeutic effects in improving sepsis-related lung injury.

### 3.3 Se and sepsis-related myocardial injury

Myocardial dysfunction associated with sepsis is associated with high morbidity and mortality rates. Sepsis can cause impaired cardiac function, arrhythmias, and hemodynamic instability [47,68]. Inflammatory responses and cytokine release result in endothelial dysfunction, leading to vasodilation and increased vascular permeability, thereby causing severe circulatory system problems such as hypotension and shock [48,68]. During acute inflammatory states and sepsis, Se levels in the blood decrease and are negatively correlated with morbidity and mortality rates [69]. Se not only contributes to myocardial injury and cardiac remodeling in heart failure [70], but Na₂SeO₃ also acts on reperfusion tissues, possibly by reducing protein carbonyl and lipid peroxide levels, thus influencing oxygen metabolism and alleviating reperfusion injury [71,72]. In a lipopolysaccharide (LPS)-induced murine sepsis model, Se pretreatment was found to markedly improve biomarkers of myocardial injury and attenuate LPS-induced cardiac dysfunction. The underlying mechanism may involve Se-mediated suppression of STING pathway activation, thereby reducing pro-inflammatory cytokine expression, alleviating oxidative stress, and inhibiting cardiomyocyte apoptosis [73]. These findings suggest that Se holds potential as a therapeutic agent for sepsis-induced cardiac dysfunction.

### 3.4 Se and sepsis-related kidney injury

Sepsis can lead to acute kidney injury (AKI), characterized by impaired renal function and decreased urine output. Mechanisms, including inflammatory response, hemodynamic changes, cellular injury, and apoptosis, collectively contribute to kidney damage in sepsis [74,75]. Se has been shown to have a protective effect against ischemia/reperfusion (I/R) injury through its antioxidant properties [76]. A study found that Se nanoparticles (NPs) prepared using Na₂SeO₃, ascorbic acid, and bovine serum albumin (BSA) significantly increased the levels of glutathione peroxidase-1 (GPx-1) in I/R cell and animal models, inhibited the activation of NLRP3 inflammasome, thereby reducing the cleavage of pro-Caspase-1 into active Caspase-1 and maturation of inflammatory cytokines, and suppressing renal inflammation. These findings suggest that Se could be a potential therapeutic agent for AKI [77]. Another similar study demonstrated that porous Se@SiO_2_ NPs provided protection against I/R-induced AKI through antioxidant and anti-inflammatory effects [78]. Furthermore, Se treatment may inhibit oxidative damage and inflammation caused by rhabdomyolysis, reduce proteinuria, and serve as a therapeutic approach for AKI [79]. Although these experimental results suggest the potential therapeutic effects of Se and its derivatives in sepsis-related kidney injury, further preclinical studies are needed to support this hypothesis.

### 3.5 Se and sepsis-related liver injury

Sepsis can lead to hepatic dysfunction and liver injury, manifested as abnormal liver function and signs of liver failure [80,81]. Inflammatory response and cellular injury contribute to hepatic inflammation, necrosis, and fibrosis, thereby affecting liver metabolism, detoxification, and synthesis functions [81,82]. Transcription and protein levels of hepatic Se enzyme SELENOP, GPx-1, and GPx-4 were significantly reduced in mice treated with LPS [69]. In a rat model of LPS/D-Galactosamine (D-GalN)-induced liver injury, curcumin (CUR) and/or Se prevented liver damage, histological changes, and oxidative stress while enhancing antioxidant defense capacity. Moreover, CUR and/or Se reduced the expression of serum CRP, pro-inflammatory cytokines in the liver, TLR4, NF-κB, JNK, and p38, and upregulated heme oxygenase-1 (HO-1). These findings suggest that CUR and/or Se mitigate liver injury in LPS/D-GalN-induced rats through inhibition of TLR4 signaling, inflammatory response, oxidative stress, and promotion of antioxidant HO-1 [83]. In a mouse model of BCG/LPS-induced liver injury, melatonin-Se NPs (MT-Se) significantly reduced the elevated serum transaminase levels, alleviated the severity of hepatocellular injury and inflammatory cell infiltration, inhibited the increase in NO levels in serum and liver tissue, and decreased the levels of tumor necrosis factor-alpha (TNF-α) and interleukin (IL)-1β in the liver [84].

### 3.6 Se and sepsis-related intestinal injury

In sepsis, infection and inflammatory response can impair intestinal mucosal barrier function [85,86]. This compromises the ability of the intestinal mucosa to prevent the passage of bacteria, toxins, and other harmful substances, potentially leading to dysbiosis, bacterial translocation, and endotoxin release [7,87]. Biogenic SeNPs effectively alleviated mitochondrial and lysosomal dysfunction in LPS-exposed IEC-J2 cells, thereby maintaining intestinal epithelial barrier integrity [88]. Another study found that SeNPs prevented oxidative stress-induced intestinal barrier dysfunction by modulating mitochondrial function and gut microbiota [89]. Recently, Se-enriched purple fractal fern (*Cardamine violifolia*, SEC) has gained attention due to its anti-inflammatory and antioxidant properties [90]. The main functional component, Se-enriched peptides extracted from purple fringed fern (*Cardamine violifolia*, CSP), improved cell viability, decreased lactate dehydrogenase activity, and enhanced cell barrier function. Mechanistically, SEC ameliorated mitochondrial dynamics disturbances induced by LPS/TNF-α in the jejunum and IPEC-1 cells. Moreover, CSP-mediated cell barrier function relied mainly on mitochondrial fusion protein MFN2 rather than MFN1. Taken together, these results indicate that SEC mitigates sepsis-induced intestinal injury through the regulation of mitochondrial fusion. Some studies suggest the presence of Se metabolism disorders and deficiencies in sepsis patients, but the exact relationship between Se and sepsis-related intestinal injury requires further investigation.

## 4 Potential of Se therapy in sepsis

The potential of Se as a treatment for sepsis-related organ dysfunction has attracted widespread attention. Preclinical and clinical studies in recent years have indicated that Se may have the potential to counteract organ dysfunction caused by sepsis.

### 4.1 Oxidative stress

The redox balance is essential for maintaining cellular homeostasis. Oxidative stress manifests as an imbalance between cellular oxidation and the antioxidant defense system. This imbalance is primarily characterized by the excessive generation of ROS that surpasses the clearing capacity of the antioxidant defense system, ultimately resulting in structural and functional damage to DNA, lipids, and proteins. Se supplementation in the diet has been shown to enhance the activity of antioxidant enzymes such as GPx and superoxide dismutase, reduce MDA levels, and decrease DNA damage and cell apoptosis caused by oxidative stress [91,92]. Se’s function in the body is mainly attributed to its antioxidant properties, as it is a crucial component of important antioxidant enzymes [93]. GPx and TrxR may reduce intracellular H_2_O_2_ and lipid hydroperoxides as part of the thiol-disulfide redox system, thus reducing oxidative stress. One of the most significant aspects of Se is its role as a constituent of many key antioxidant compounds and its unique oxidative property as an antioxidant molecule, thiol peroxidase. GPX protects membrane integrity by reducing ROS metabolites [94]. Se also plays a critical role as an antioxidant in anti-aging processes [95]. Importantly, both Se salts and organic Se compounds have been shown to effectively improve oxidative stress in animal models of sepsis and in multiple clinical trials involving sepsis patients [96].

### 4.2 Immune system regulation

Numerous studies have demonstrated that Se supplementation enhances the immune response to various harmful conditions [26,34,35]. Se supplementation is involved in the regulation of innate and adaptive immunity [97]. It has a significant immunostimulatory effect, including T-cell proliferation, natural killer cell activity, and innate immune cell functions [34]. Studies have found that increased Se intake upregulates ribosomal protein and translation factor gene expression and enhances lymphocyte function [35]. Additionally, Se can block the expression of NK cell inhibitory receptors, thereby activating NK cell immune capabilities. *In vitro* and *in vivo* experiments have revealed potential chemoenhancement and immune activation mechanisms of Se, highlighting its prospects in immune therapy [36].

### 4.3 Regulation of inflammatory response

Se and selenoproteins have significant anti-inflammatory effects in various diseases, particularly during the wound healing process [16,98]. Se and selenoproteins exert protective effects on immune responses and epithelial barrier integrity after intestinal infections by regulating ILC3 and Th17 cells, thus alleviating inflammation and infection [99,100]. In the cardiovascular system, selenoproteins may protect endothelial cells by reducing abnormal cell adhesion induced by pro-inflammatory cytokines [101,102]. The nuclear factor kappa-B (NF-κB) signaling pathway is associated with enhanced inflammatory responses, and its activation is significantly correlated with the production of IL-6 and TNF-α. Se may inhibit the activation of NF-κB by modulating the expression of selenoprotein genes. Additionally, Se supplementation in chronic inflammation can restore depleted liver and serum Se levels by increasing the biosynthesis of selenoproteins, thereby inhibiting the production of CRP and alleviating the inflammatory process [16]. Se-binding protein 1 (SELENBP1) is elevated in sepsis, and its deficiency prolongs survival in mice, alleviates hepatic injury, and reduces inflammation, while increasing the splenic Treg/Th17 ratio. Mechanistically, SELENBP1 deficiency suppresses dendritic cell (DC) maturation, promotes the development of a tolerogenic DC phenotype, and regulates immune homeostasis. These findings indicate that SELENBP1 participates in the progression of sepsis by modulating DC immunoactivity, thereby providing a potential therapeutic target [103].

### 4.4 Regulation of cell death

Se regulates cell death through various mechanisms, including apoptosis (programmed cell death) and necrosis (unprogrammed cell death). Low serum levels of α-tocopherol and Se are associated with accelerated apoptosis during severe sepsis [104]. Se not only protects against GPx4-mediated lipid peroxidation and apoptosis in germ cells [105] but also improves excessive cell apoptosis in various muscular disease models [106,107].

Furthermore, the Se-dependent enzyme GPX4 is a key regulatory factor in ferroptosis [108]. For instance, Se supplementation can enhance the expression of GPX4, increase the number of follicular helper T cells, and modulate gene transcription programs, thereby inhibiting lipid peroxidation and protecting cells from ferroptosis-associated iron defects [109]. Additionally, Se plays a regulatory role in processes such as autophagy [110–112] and pyroptosis [113,114].

## 5 Se in clinical trials for sepsis treatment

In exploring the potential of Se as a treatment for sepsis, several clinical trials have been conducted or are currently underway. These trials aim to evaluate the efficacy and safety of Se therapy in sepsis patients, providing stronger evidence for clinical practice.

Critically ill sepsis patients have lower Se levels [22], and concentrations below 0.7 μmol/L are associated with higher mortality rates and organ dysfunction in ICU patients [115]. In a clinical trial conducted by Angstwurm et al. [52], sepsis or septic shock patients with an APACHE III score >70, were administered 1 mg of Na₂SeO₃ followed by a continuous daily infusion of 1 mg for 14 days, resulting in a decreased 28 days mortality rate compared to the placebo group, with sufficient levels of Se and GSH-Px-3 in the serum. Kocan et al. demonstrated that adjunctive therapy with a continuous dose of 750 µg/24 h of Na₂SeO₃ was beneficial for patients with sepsis-associated acute lung injury [116]. The effectiveness of Se therapy in severe sepsis or septic shock remains controversial. A systematic review and meta-analysis of 13 randomized controlled trials (RCTs) found that there was no association between Se treatment and a decrease in mortality rates at different time courses. However, supplementation with Se did not show significant efficacy in terms of renal failure incidence, secondary infections, or duration of mechanical ventilation. Se therapy was beneficial for sepsis patients as it reduced the duration of vasopressor therapy, shortened the length of stay in the intensive care unit and hospital, and decreased the incidence of ventilator-associated pneumonia [117]. In a secondary analysis of the SISPCT RCT, 1,089 patients with severe sepsis or septic shock were classified into four phenotypes (α, β, γ, δ) based on the SENECA cohort. The results showed slight differences in 28 days mortality among the phenotypes (α 20.8%, β 20.3%, γ 27.1%, δ 28.5%), but these differences were not statistically significant. High-dose intravenous Se conferred no significant benefit in any phenotype [19]. In sepsis, neutrophil respiratory burst contributes to endothelial injury. Plasma antioxidant selenoenzymes, including SePP and GPX3, are decreased, whereas Na₂SeO₃ can serve as a Se donor or act as a concentration-dependent oxidizing agent. A multicenter, double-blind study demonstrated that continuous infusion of Na₂SeO₃ increased plasma Se, SePP, and GPX3 levels; however, no significant improvements were observed in organ function, blood lactate levels, or survival outcomes, and no toxicity was reported. These findings suggest that the clinical benefits of Se supplementation remain unclear and warrant further investigation [118]. Studies on critically ill patients have shown conflicting results, possibly due to different patient populations, study designs, timing, dosing regimens, intervention durations, and outcome measures assessed [22]. Therefore, it is necessary to strengthen the evidence on these indicators by focusing on specific patient populations and using more comparable study protocols.

Although more research is still needed to determine the exact role of Se therapy in sepsis, the ongoing clinical trials provide hope in uncovering the potential of Se as a therapeutic strategy. These trials are expected to offer more meaningful treatment recommendations to clinicians for improving the prognosis and survival rates of sepsis patients. Additionally, there are several multicenter, randomized controlled trials currently underway or being planned, aiming to further evaluate the role of Se therapy in sepsis. These trials will involve larger sample sizes, compare the effects of Se treatment with standard therapy or placebo, and assess its impact on inflammatory response, organ function, and survival rates.

## 6 Safety and limitations

The potential of Se as a treatment for sepsis has garnered significant attention. However, when considering its clinical application, we must recognize its safety and certain limitations. Se, as an essential trace element, plays a crucial role in normal physiological functions. Nevertheless, it is important to note that excessive intake can lead to Se toxicity. Therefore, ensuring a reasonable dosage when supplementing Se is crucial for patient safety. Additionally, the side effects and adverse reactions of Se therapy also warrant attention. While Se is widely regarded as a relatively safe supplement, adverse reactions such as nausea, vomiting, and diarrhea may occur in specific individuals and circumstances [119,120]. Thus, close monitoring of patients’ clinical symptoms and responses, along with dose adjustments and, if necessary, discontinuation of treatment, should be implemented when supplementing Se.

In clinical research, exploration is underway regarding the administration routes, dosages, and timing of Se. Studies have shown that oral Se supplements, intravenous Se injections, and Se-rich nutritional support can effectively replenish Se levels [121–123]. However, the selection of dosage and timing should be individualized based on the patients’ clinical characteristics and condition.

Sepsis is a complex disease involving disruptions and dysfunctions in multiple organ systems. Se therapy may only be a part of comprehensive sepsis management rather than a standalone treatment approach. Therefore, Se therapy should be integrated into a comprehensive treatment strategy, combining other therapeutic measures such as antibiotic therapy, vasopressors, and fluid resuscitation. Finally, the indications and timing for Se therapy need further clarification. Currently, Se therapy has not been widely incorporated into clinical guidelines for sepsis. Therefore, clinicians should carefully weigh the potential benefits and risks of Se therapy and make decisions based on individualized circumstances. Notably, recent studies have proposed novel strategies to enhance the efficacy of Se and its combination therapies. For example, calycosin (CA), one of the major active flavonoids in Astragalus, exhibits therapeutic potential in sepsis, but its low plasma concentration and poor bioavailability limit clinical effectiveness. Loading CA into BSA-coated SeNps (BSA@Se-CA, BSC) significantly enhances its ROS scavenging capacity and GPX activity, while synergistically inhibiting the NF-κB signaling pathway to reduce the expression of inflammatory mediators, including NO, IL-6, IL-1β, and TNF-α. *In vitro*, BSC protects the function of RAW264.7 and HUVEC cells; *in vivo*, intraperitoneal injection of BSC increases survival and alleviates organ injury in septic mice [124]. In addition, sepsis-associated acute lung injury (ALI) remains a major clinical challenge. Bone marrow-derived mesenchymal stem cells (BMSCs) possess anti-inflammatory and cytoprotective properties, but their function declines with age. Functionalization of BMSCs with chitosan-coated SeNPs promotes selenoprotein biosynthesis via miR-20b and enhances mitochondrial transfer, delivering functional mitochondria to injured alveolar epithelial cells for repair. Simultaneously, this approach suppresses the RORγt/STAT3/Th17 pathway, reducing CD4⁺ T cell differentiation into Th17 cells. This dual mechanism of immune modulation and mitochondrial repair significantly decreases inflammatory markers in mouse models and outperforms conventional BMSC therapy, offering a nano-enabled stem cell strategy for sepsis-induced ALI [125]. In summary, Se and related nanocomposites show promising potential in sepsis therapy. However, their clinical application requires careful consideration of safety, dosage, administration route, and timing, ideally in combination with integrated therapeutic strategies. Future studies and clinical trials will be essential to further evaluate efficacy and guide clinical translation.

## 7 Future outlook

The potential of Se as a treatment for sepsis has generated widespread interest, and future research and prospects will further drive the development in this field. First, further clinical studies are necessary to evaluate the long-term effects and safety of Se therapy in sepsis. Large-scale, multicenter, randomized controlled trials will not only help determine the optimal dosage, administration routes, and timing of Se therapy but also further assess the impact of Se therapy on clinical outcomes in sepsis patients, such as survival rates, organ function recovery, and hospital stay duration.

Additionally, the mechanisms of Se in sepsis deserve in-depth investigation. For example, a comprehensive study of Se’s antioxidant stress, anti-inflammatory, and cellular protective mechanisms is crucial for optimizing its therapeutic effects. Further preclinical research can elucidate the interactions between Se and the pathological processes of organ dysfunction in sepsis and help unravel the molecular and cellular mechanisms of its actions. Moreover, from the perspective of personalized treatment and precision medicine, analyzing patients’ genotypes, phenotypes, and disease characteristics can identify those who are most likely to benefit from Se therapy and develop individualized treatment plans. This undoubtedly contributes to improving treatment efficacy and reducing the risk of adverse reactions in patients. Finally, leveraging technologies such as artificial intelligence, machine learning, and data mining, the analysis of large-scale clinical data can discover potential indicators of Se therapy, predict patients’ treatment responses, and optimize treatment strategies.

## 8 Conclusion

In conclusion, Se exhibits potential mechanisms and therapeutic prospects in treating organ dysfunction associated with sepsis. Through further clinical research, mechanistic investigations, personalized treatment, and the application of intelligent technologies, we can better understand the mechanisms of Se therapy, optimize its application, and ultimately improve the prognosis of sepsis patients.
